# A modern mold room: Meshing 3D surface scanning, digital design, and 3D printing with bolus fabrication

**DOI:** 10.1002/acm2.12703

**Published:** 2019-08-27

**Authors:** David Kiyoshi Sasaki, Philip McGeachy, Jorge E. Alpuche Aviles, Boyd McCurdy, Rashmi Koul, Arbind Dubey

**Affiliations:** ^1^ Department of Medical Physics CancerCare Manitoba Winnipeg Manitoba Canada; ^2^ Department of Medical Physics Tom Baker Cancer Centre Calgary Alberta Canada; ^3^ Department of Physics and Astronomy University of Manitoba Winnipeg Manitoba Canada; ^4^ Department of Radiology University of Manitoba Winnipeg Manitoba Canada; ^5^ Department of Radiation Oncology CancerCare Manitoba Winnipeg Manitoba Canada

**Keywords:** 3D printing, 3D scanning, bolus, digital design, mold room

## Abstract

**Purpose:**

This case series represents an initial experience with implementing 3‐dimensional (3D) surface scanning, digital design, and 3D printing for bolus fabrication for patients with complex surface anatomy where traditional approaches are challenging.

**Methods and Materials:**

For 10 patients requiring bolus in regions with complex contours, bolus was designed digitally from 3D surface scanning data or computed tomography (CT) images using either a treatment planning system or mesh editing software. Boluses were printed using a fused deposition modeling printer with polylactic acid. Quality assurance tests were performed for each printed bolus to verify density and shape.

**Results:**

For 9 of 10 patients, digitally designed boluses were used for treatment with no issues. In 1 case, the bolus was not used because dosimetric requirements were met without the bolus. QA tests revealed that the bulk density was within 3% of the reference value for 9 of 12 prints, and with more judicious selection of print settings this could be increased. For these 9 prints, density uniformity was as good as or better than our traditional sheet bolus material. The average shape error of the pieces was less than 0.5 mm, and no issues with fit or comfort were encountered during use.

**Conclusions:**

This study demonstrates that new technologies such as 3D surface scanning, digital design and 3D printing can be safely and effectively used to modernize bolus fabrication.

## 1 Introduction

Traditional mold rooms have been used in radiation therapy for decades to design and fabricate the various devices required for radiation treatment including immobilization, shielding, and bolus. For bolus fabrication this traditionally entailed use of technologies such as plaster of Paris, beeswax, and synthetic gel sheets. The process required a dedicated clinical space, the physical presence of the patient for key steps of the design and fabrication process, physical contact with potentially sensitive areas, and significant manual labor. We have recently developed a new approach where digital scanning, specialized 3D software, and 3D printing have been used, along with a novel Quality Assurance (QA) procedure designed to ensure the new technologies are used safely. This paper documents the first case series of external beam bolus fabricated using this process at our center.

While 3D surface scanning technology is not new, low‐cost 3D scanners suitable for use in radiotherapy have only recently become available. To date a handful of authors have investigated their use for patient position monitoring,^1^ improving the extended field‐of‐view in computed tomography (CT)^2^ and total body irradiation (TBI) compensator design.^3^ We have previously documented the use of this technology to help streamline our lead shielding fabrication process for orthovoltage treatments.^4^

Low‐cost three‐dimensional (3D) printing is also a relatively new technology that is currently being used in radiation therapy. In the literature this technology has been used to fabricate compensators for TBI,^5^ brachytherapy applicators,^6^, ^7^ range compensators for proton therapy,^8^, ^9^ and phantoms for quality assurance.^10^, ^11^ Various investigators have utilized 3D printing for bolus fabrication for megavoltage photon and electron treatments as well,^8^, ^12^, ^13^, ^14^, ^15^, ^16^ but only a few describe a full case series.^17^, ^18^, ^19^

There are a number of different 3D printing technologies currently available, an overview of which is provided elsewhere.^9^, ^10^ Fused deposition modeling (FDM) is the most commonly used technology due to its low capital and material costs. Fused deposition modeling printers extrude thin strands of melted plastic and fuse them to previously deposited strands. In this way an object is built up layer by layer. A FDM printer can typically print with a number of materials, but polylactic acid (PLA) and acrylonitrile butadiene styrene (ABS) are commonly used in radiotherapy as they are reasonably water equivalent.^9^, ^15^ Both materials are rigid when printed, and this presents a challenge—in order for a treatment device to fit comfortably, the finished piece must closely reproduce a patient’s surface contours or pressure points will result.

There are three properties that a 3D printed bolus must exhibit in order to fulfill its purpose: it must have a uniform and reproducible bulk density and the final shape must closely match the design, especially if the material is rigid. Due to the nature of FDM printing, these properties can vary from print to print^20^ so a thorough method of quality assurance was developed.

This work presents our experience in using 3D surface scanning, 3D modeling software, and 3D printing in fabricating bolus for the first 10 patients who required customized bolus in a region with complex contours. This process used equipment that has little cost and software that is either free or easily accessible. The process could therefore be easily adopted.

## 2 Methods and Materials

### 2.1 Patients

Clinical implementation of digital scanning, design, and fabrication of bolus began at our center in 2016. In this study, medical physicists and radiation oncologists identified 10 patients for whom digital design and fabrication would be superior to conventional bolus fabrication based on the location of the tumor and the complexity of surface contours. Clinical details of the 10 patients are listed in Table 1. The average age at diagnosis was 68.4 yr. The majority of patients (50%) had a diagnosis of basal cell carcinoma of the skin and two patients had a diagnosis of plasmacytoma. Seven patients had superficial tumors involving the skin. Different dose and fractionation schedules were used depending on the pathology. Six patients were treated with megavoltage photons and the remaining four were treated with electrons. An optical surface scan was acquired in three of seven superficial tumors for generating a 3D printed bolus; for the remaining patients CT scan data were used. Interdisciplinary discussions were held between the radiation oncologist, physicist, therapist, and dosimetrist prior to defining the extent and thickness of bolus. For patient 1, the 3D printed bolus was not used even though the bolus was successfully printed, since the dosimetric requirements were met without the bolus.

**Table 1:** Treatment and patient information for the first patients to receive 3D printed bolus

Patient #	Age (yrs)	Sex	Site	Pathology	Stage	Intent	RT dose (Gy/Fx)	RT energy	RT modality	Scan type
1	61	M	Nasal Cavity	Plasma‐cytoma	NA	R	46/23	6 MV	Ph	CT
2	67	F	Knee	BCC	T3N0	P	36/6	6 MV	Ph	CT
3	72	M	Ear	BCC	T2N0	R	55/22	12 MeV	E	O
4	55	M	Scalp	Myeloid Sarcoma	NA	P	30/15	12 MeV	E	CT
5	63	F	Nose	BCC	T1N0	R	48/15	9 MeV	E	O
6	62	M	Nose	BCC	T2N0	R	45/15	9 MeV	E	O
7	82	M	Tibia	Plasma‐cytoma	NA	R	50/25	6 MV	Ph	CT
8	69	F	Nose	BCC	T1N0	A	55/22	6 MV	Ph	CT
9	84	M	Scalp	SCC	T2N0	R	55/20	6 MV	Ph	CT
10	69	F	Lacrimal Gland	FL	IAE	R	24/12	6 MV	Ph	CT

Abbreviations: A, Adjuvant; BCC, Basal cell carcinoma; E, Electrons; F, Female; FL, Follicular lymphoma; M, Male; NA, Not applicable; O, Optical scan; P, Palliative; Ph, Photons; R, Radical; SCC‐Squamous cell carcinoma.

### 2.2 3D Scanner

A structured light scanner that employs an infrared light source and camera, along with an optical camera to capture color information (Sense 3D Scanner, 3D Systems, USA) was used in this work. We have previously described the commissioning, operation and accuracy of this device.^4^ As the intention of this project at our center was to increase efficiency, the optical scanner was used whenever possible as it can be operated by only one therapist in a standard examination room. The scanner has limitations that prevented its use on all patients, however. These limitations are.

1. The scanner can only reconstruct surfaces to which it has a direct line of sight, and therefore any treatment area with folds or overlapping anatomy cannot be fully reconstructed.
2. Only the surface anatomy is reconstructed, so in cases where the extent of the bolus required is uncertain without visualization of underlying tissue another imaging modality is needed.
3. Hair is generally reconstructed as a solid surface by the scanner and is therefore problematic, unless it is very thin or is a good approximation of the true patient surface (eg, the eyebrows). For areas like the scalp, where the removal of overlying thick hair may be undesirable, the optical scanner cannot reconstruct the true patient surface.

CT imaging was used for cases where the above limitations precluded the use of the optical scanner. There were two differences in workflow using CT vs optical scan data: (a) MeshMixer was the only option for bolus design for patients who received optical scans and (b) Step artifacts were not present in optical scans, saving a mesh processing step.

### 2.3 3D Design and analysis software

**Figure 1:**
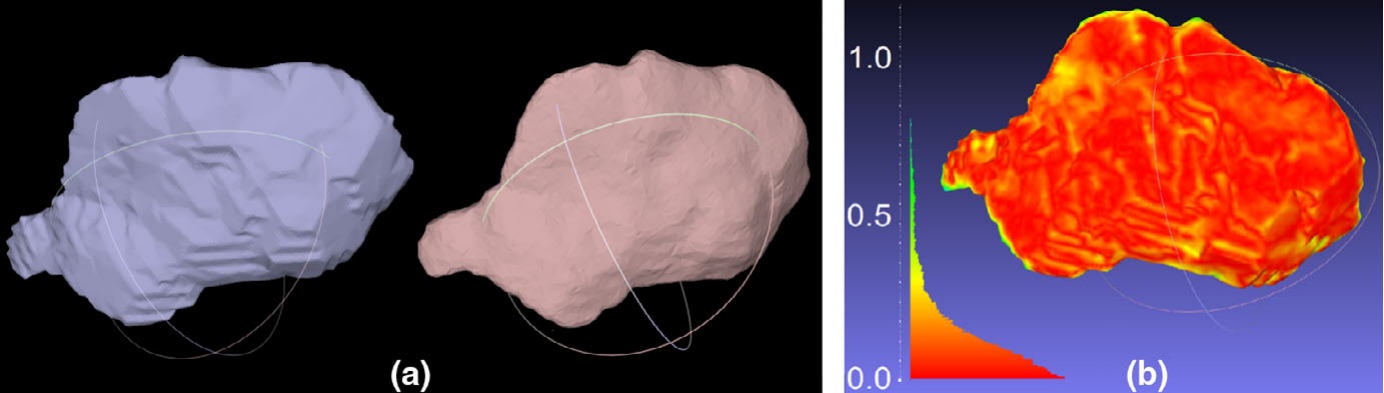
Post processing boluses created in Eclipse. a) A side by side comparison of an original bolus exported from the Eclipse TPS (left) and the smoothed bolus (right). Note that the step artifacts resulting from the 3 mm slice spacing are no longer apparent in the smoothed mesh. (b) For demonstration purposes, a detailed comparison between the two meshes is shown, illustrating that differences due to smoothing are negligible dosimetrically. The legend is in millimeters. The above analysis was not done routinely—a quicker method of verification was employed using a thickness measurement tool over the surface of the processed bolus. TPS, treatment planning system.

The Eclipse treatment planning system (TPS) (Varian Medical Systems, USA) was used to design boluses for those patients who received CT scans for contour acquisition and whose bolus was a simple expansion of a volume (eg, the body). MeshMixer (Autodesk, USA) was used for design for patients who received optical scans or when the desired piece was complex. MeshMixer was also used to postprocess boluses designed in Eclipse, for example, to smooth edges caused by the 3 mm CT slice spacing. As this post processing is a potential source of error, it is explained in some detail. The post processing is illustrated in Fig. 1, along with a quantitative comparison between the original and processed bolus. If the patient was not re‐scanned with the printed bolus in place, extra measurements were performed on the processed bolus to ensure that no significant differences between the planned and actual dose distributions were introduced, often using a thickness measurement tool inside MeshMixer. STL files were converted to printer instructions using Repetier‐Host (Hot‐World GmbH & Co., Germany). MeshLab, an application for mesh analysis (MeshLab 64 bit v1.3.3, Visual Computing Lab – ISTI – CNR, http://meshlab.sourceforge.net/) was used for the QA process.

**Figure 2:**
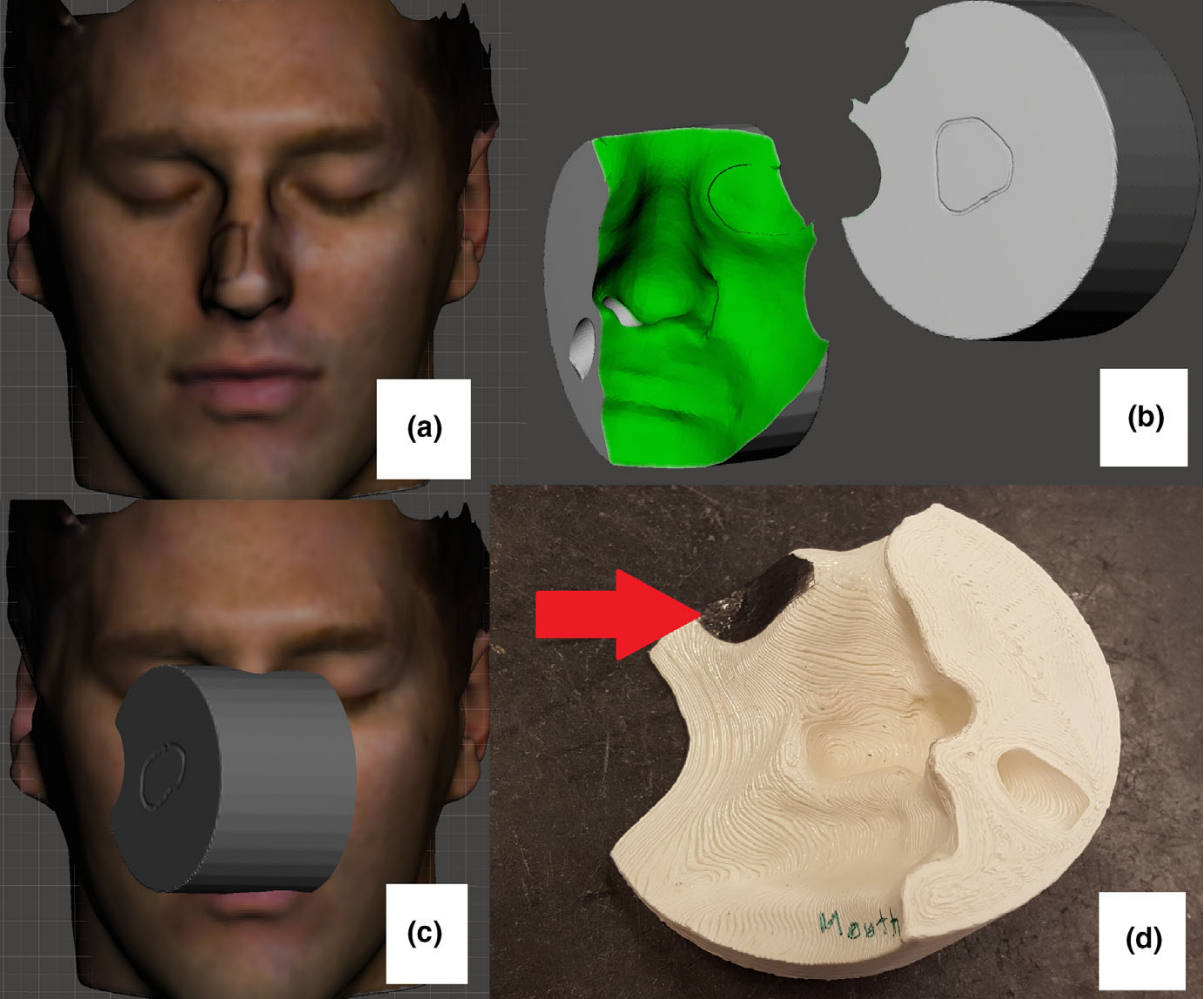
The design process for an electron bolus. a) Optical scan of volunteer for MeshMixer bolus design. b) Design, including flat surface on beam entrance side, breathing tunnel and eye shielding recess. c) Treatment geometry. d) printed bolus, with lead shielding in place (red arrow).

Four of 10 pieces were designed using Eclipse, and four were designed using MeshMixer. In two cases both were used. Patients 3‐6 required an electron bolus that matched the contours of the patient on one side and was flat on the other. The easiest way to design these pieces in MeshMixer is to start with a basic shape such as a cylinder, align it with the patient external contour, and use Boolean operations to yield a customized bolus. These bolus pieces also had the field shape engraved onto the flat side of the bolus for setup purposes. Patients 5 and 6 had electron boluses designed with recesses over the patient’s ipsilateral eyes to accommodate 2 mm lead shielding, resulting in a combined bolus/shielding device. Breathing tunnels for the contralateral nostrils were also included. The design process is shown in Fig. 2.

**Figure 3:**
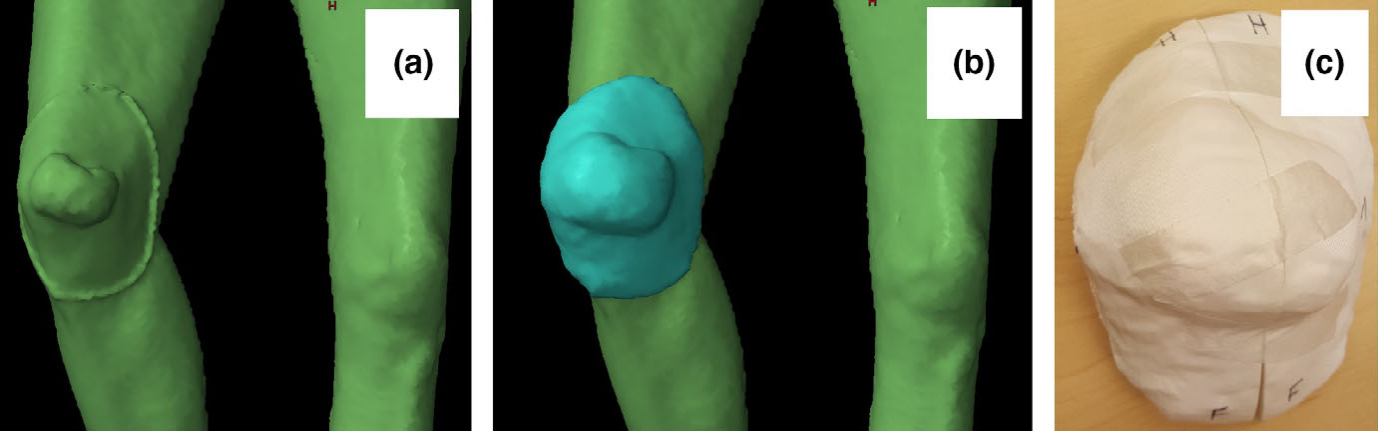
Design process for a photon bolus. a) Knee lesion with affected skin marked with wire. b) Bolus designed in Eclipse before processing with MeshMixer. c) Printed bolus.

For patient 2, both Eclipse and MeshMixer were used during design. The bolus was designed for a knee lesion that had a protruding, hemispherical component, with extension to the skin around the lesion (Fig. 3). The bolus was designed in two pieces that could be fit together over the knee with a seam that ran perpendicular to the plane of gantry motion during a RapidArc treatment. A gap was designed into the bolus to allow for a 2 mm space between the bolus and main lesion to avoid physical contact, both to increase patient comfort and to allow for any swelling that might occur.

## 3 Treatment Planning

### 3.1 Electrons

At our center, electron treatments are reserved for simple cases. We do not use the treatment planning system for dose calculations—these are done by hand using water tank data. The purpose of the bolus in this case is to get the treatment geometry as close as possible to a water tank, and provide the appropriate buildup. In these cases the physical thickness of the bolus is scaled according to the measured density of the material found during commissioning, and the quality assurance process outlined below is used to verify this assumption.

### 3.2 Photons

For more complicated skin lesions (eg, ones that are very large), or for deeper tumors that extend to the surface, photons are used. In these cases the bolus is either created before the planning CT scan using either CT or optical scan data, or designed in Eclipse on the planning CT scan using the bolus tool and exported for printing. When the bolus is designed in Eclipse, the Hounsfield units (HU) of the bolus are assigned a value such that the bolus density is equal to that found during commissioning. As with electron boluses, the QA process outlined below is used to verify the assumed density.

### 3.3 Printer and filament

All boluses presented in this paper were fabricated using a MakerGear M2 printer (MakerGear, USA) and ColorFabb PLA/PHA filament (ColorFabb, The Netherlands). The polymer used in this filament has a bulk density of 1.24 g/cm^3^. The print settings used for the majority of prints can be found in Table 2.

**Table 2:** General print settings used for the boluses in this work

Bed temp	65°C	Perimeter speed	~40 mm/s
Nozzle temp	205°C	Perimeters	3
Bottom solid layers	3	Small perimeter Speed	~15 mm/s
Brim width	15 mm	Solid infill Speed	~40 mm/s
External fill pattern	Concentric	Support material angle	30°
External perimeter speed	50%	Support material contact distance	0.2 mm
Fill angle	45°	Support material enforce layers	3
Fill density	100%	Support material interface layers	3
Fill pattern	Rectilinear, Concentric	Support material pattern	Rectilinear grid
First layer extrusion width	175%	Support material spacing	2.5 mm
First layer height	0.5, 0.3 mm	Support material speed	~30 mm/s
First layer speed	~10 mm/s	Top solid infill speed	~15 mm/s
Infill speed	~40 mm/s	Top solid layers	3
Layer height	0.5, 0.3 mm	Nozzle diameter	0.5, 0.35 mm
Max print speed	80 mm/s		

Before first clinical use, the printer was commissioned by printing rectangular slabs of PLA and measuring their physical characteristics such as dimensional accuracy, density, and attenuation in a manner similar to that which has been previously reported.^9^, ^15^ For QA purposes, these slabs were also imaged using CT in order to assess their HU characteristics, and to find a CT number threshold for autosegmentation that would yield dimensions that matched those measured with calipers. The reference values we collected were: printed physical density of 1.2 g/cm^3^, average CT number of 120 HU, standard deviation in CT number of 12 HU, and a CT number threshold of −300 HU for identifying the physical surface boundary of the slabs. The hydrogel sheet used for custom bolus at our center was also imaged using CT to assess its density uniformity. The standard deviation in the CT number for the hydrogel sheet was 59 HU, and this was taken as an initial uniformity tolerance.

### 3.4 Quality assurance process

For quality assurance, all 3D printed boluses were imaged using x‐ray CT (120 kVp, 350 mAs, 0.4 mm slice spacing, 0.8 mm slice thickness, 180 or 250 mm field‐of‐view). Images were imported into Eclipse where the external contour of the bolus (EXT) was autosegmented using the −300 HU threshold. A script was used to export the EXT contour in polygon file format (EXT PLY). A second contour was generated by contracting EXT by 1 mm uniformly to exclude any voxels affected by the partial volume effect (EXT CONTRACT). A second in‐house script was used to export the CT number of every voxel inside the EXT CONTRACT contour into a text file. These text files were analyzed using Matlab (The MathWorks, Inc, USA). The mean, median, standard deviation, minimum and maximum CT numbers were calculated, and a histogram was generated.

The external contour in mesh format (EXT PLY) was then compared to the original design mesh (DESIGN). Both meshes were imported into MeshLab and were aligned with each other in a two‐step process. The first step was a manual landmark registration, the result of which was used as the input to an iterative closest point (ICP) algorithm.^21^, ^22^ After each run of the ICP algorithm, the average and median error is reported and the registration was repeated until the values no longer changed. Once aligned, we then applied an algorithm in MeshLab described by Cignoni et al.^23^ that calculates the distances between the two aligned meshes over the entire surfaces. Briefly, the surface of EXT PLY was sampled thousands of times. The distance between each sampled EXT PLY point and the nearest location in DESIGN was found. These distances represent shape errors resulting from the printing process. The errors can be displayed as a colorwash on EXT PLY or DESIGN, and error statistics and histograms can be generated. We recorded the mean, median, standard deviation, maximum and minimum errors found, along with shape error histograms. This analysis took roughly 10 minutes per print, not including CT imaging.

The final QA test was the fit of the bolus on the patient, performed at first treatment fraction. For this initial series of patients, medical devices staff members (ie, machinists) were on standby in case any alterations were required. Fit to patient was assessed visually by the therapists, and using feedback from the patients regarding any discomfort.

## 4 Results

### 4.1 Quality assurance

**Figure 4:**
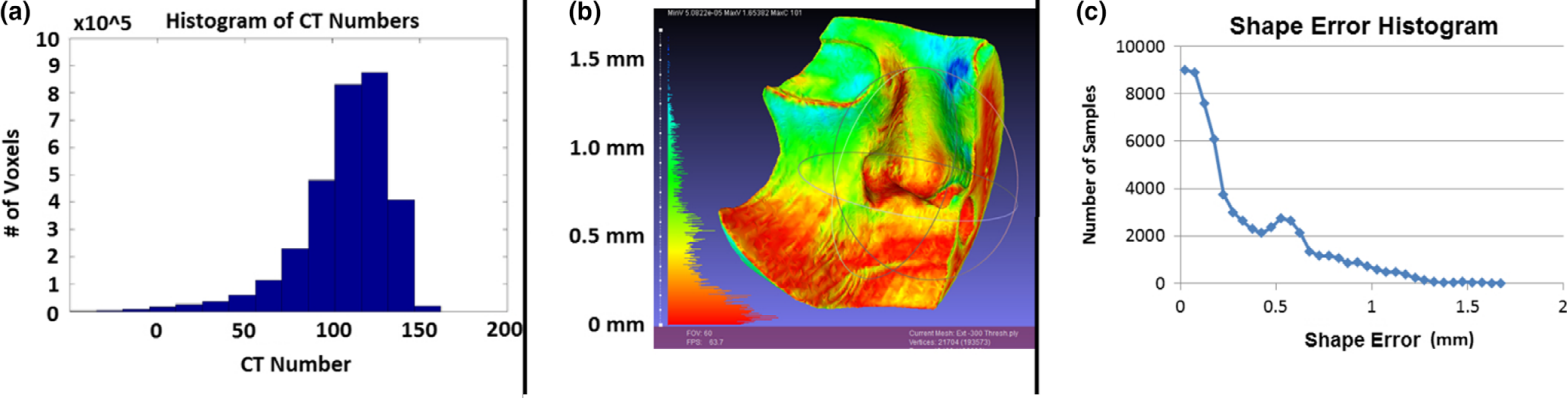
Full QA results for patient 5. a) density uniformity of the 3D print in terms of CT number. b) and c) colorwash and histogram of shape errors between the final print and the original design. QA, Quality Assurance.

As an example of the QA analysis used at our center, QA results from patient 5 are shown in Fig. 4.

From the CT number histogram in [Fig. 4(a)] we see that the modal CT number is close to the expected value of 120 HU, with a tail that extends down past 0 HU. This histogram shape is typical of the bolus prints. The tail arises from very small (<1 mm diameter) pockets of air that result from imperfect filling during printing, usually where two different fill patterns join together. These regions are spread throughout the volume of the bolus. From the shape error color wash in [Fig. 4(b)], we can easily see areas where the print deviates from the design. In this case the area of largest discrepancy (the blue region by the contralateral eye) was caused by an unsupported overhang, and although clinically insignificant as assessed at first fraction, this finding was used to improve subsequent prints. When required, this visualization can be used to manually correct a print before first use (eg, using a heat gun). The shape error histogram shown in [Fig. 4(c)] gives a useful overview of the shape accuracy: in this case, despite the complex shape of the design, the average deviation was 0.33 mm.

The QA results for all 12 boluses are shown in Table 3.

**Table 3:** QA results for 12 3D printed boluses

Patient #	Bolus #	CT Number (HU)						Shape errors (mm)			
		Mean	ABS(δ)	Median	Max	Min	St Dev	Mean	Median	Max	Min
1	1	123	3	125	155	‒49	14	0.303	0.219	1.21	0
2	2	138	18	142	205	‒290	24	0.425	0.284	3.44	0
2	3	133	13	138	191	‒300	25	0.401	0.253	3.59	0
3	4	118	2	125	202	‒299	35	0.33	0.163	2.11	0
4	5	124	4	127	211	‒160	32	0.356	0.227	2.26	0
5	6	108	12	113	162	‒141	26	0.328	0.21	1.65	0
6	7	147	27	158	211	‒159	37	0.287	0.172	1.6	0
7	8	35	85	46	174	‒502	79	0.314	0.224	3.41	0
7	9	13	107	31	167	‒522	92	0.293	0.196	2.26	0
8	10	287	167	300	423	‒582	65	0.252	0.195	1.49	0
9	11	140	20	142	173	79	9	0.238	0.187	2.02	0
10	12	147	27	148	188	57	12	0.264	0.206	1.92	0
Elastogel		‒7		‒7	160	‒188	59	–	–	–	–

ABS(δ) represents the absolute difference between the mean HU and the expected value. The red shaded values deviated more from the reference commissioning slabs than their counterparts, and so prompted further investigation.

**Figure 5:**
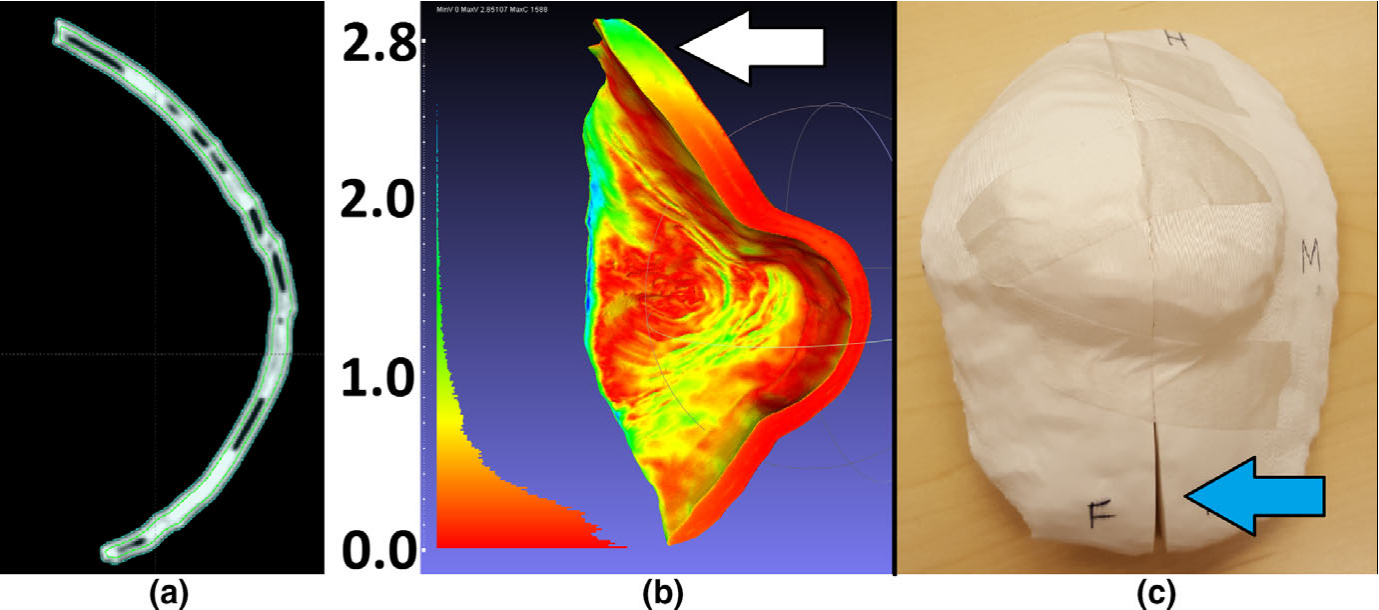
Bolus QA results of interest in the first 10 patients. a) The voids resulting from attempting to print a thin shell with a large nozzle diameter and large extruded width. These voids caused the average CT number to deviate from the expected value. b) A discrepancy due to warping in a flat surface. This resulted in a gap between two bolus pieces that were supposed to fit together, as shown in c). QA, Quality Assurance.

From the table we see that the mean CT number of the bolus pieces was within 30 HU of the reference value obtained during commissioning for the majority of the bolus pieces, which equates to 3% in bulk density based on the CT number calibration curve used in our TPS. Three prints exhibited means that were outside this boundary—those for patients 7 and 8. The two prints for patient 7 were 5 millimeter shells printed with a larger extruder diameter (0.5 mm) and a larger layer height (0.5 mm) for speed. This led to a thin area in the middle of the shell that the printer could not concentrically infill due to the relative size of the infill region compared to the extruded diameter [Fig. 5(a)]. In the case of patient 8, the printer was extruding filament at a faster rate than usual during printing, visually evident from the rough finish of the piece. Precisely why this occurred remains unknown. Since the printed boluses are mixtures of plastic and air, the increased extrusion led to an increased density. Neither of these issues was clinically significant, as assessed by re‐calculating the plan with a bolus assigned a density matching that found during QA. Apart from the three cases with deviations in mean CT number already discussed, the standard deviation for the prints was smaller than for our traditional bolus material.

The mean shape errors were less than 0.5 mm in all cases. The maximum values ranged from 1.2 mm to 3.6 mm, but this value was not useful in identifying potential fitting issues since it could occur on surfaces that were neither in contact with the patient nor in the beam path. The errors occurring on these surfaces were visually identified using the color wash. Figures 5(b) and 5(c) illustrate this point for patient 2. In [Fig. 5(b)] a 1.5 mm error due to warping is visible on the flat portion of the bolus. This occurred on both pieces. The result was a gap of 2–3 mm in the final assembly, as seen in [Fig. 5(c)]. Subsequent prints with a similar shape were printed with extra support at the base to allow for better adhesion between the first layer and the printing bed.

### 4.2 Treatment

There were no significant issues reported by the physicians, physicists or radiation therapists when using this new design and fabrication process for bolus. None of the patients reported any discomfort in using the bolus, and visual inspection by the therapists qualitatively confirmed that the pieces conformed well to the patient surfaces. No bolus pieces required adjustment during treatment.

## 5 Discussion

In this work, a case series is presented of the first 10 patients at our institute whose boluses were fabricated using a digital process for a variety of sites and pathologies, and with both high energy electron and photon beams. The use of the digital process allowed for the design of complex pieces and automated fabrication.

While the rigidity of the boluses might seem to be a weakness, it proved to have advantages. Bolus placement was simple and quick since the bolus could only fit onto the patient in one orientation. In some regions the bolus also acted as a setup/immobilization device, since it would only fit if the simulation setup was reproduced. The rigidity also allowed for the insertion of lead shielding forming a combined bolus/shielding accessory.

A novel QA method used to ensure safe and effective implementation of the new digital fabrication process has been presented. The QA method uses CT imaging to assess local and bulk density and provides thorough, quantitative assessment of shape accuracy. The methods and data may be useful for others looking to adopt 3D printing. In our patients there were no issues that required boluses to be altered. The process can be used to diagnose problems with an accessory before first use and for ongoing improvement. An advantage of this technique over other published methods is that the shape is evaluated more thoroughly, giving high confidence of a good fit. We therefore do not routinely use verification imaging or require planning simulation with the finished accessory in place, thereby streamlining the treatment process.

It is worth pointing out the limits to the QA process presented. Sources of uncertainty would include the resolution of the CT scanner (voxel size of roughly 0.5 mm), the thresholding used to define the external of the printed bolus and the registration between the design and measured external contour meshes (whose magnitude is likely dependent on the bolus shape). Taken together, we estimate an uncertainty on the order of < 1 mm. While this is inadequate to characterize the accuracy of many printers, it is sufficient to detect print errors (eg, those due to thermal warping) and to ensure the clinical accuracy of a bolus. While there are methods by which the true accuracy of printers could be assessed using metrology techniques familiar to most machine shops, the equipment required would be expensive and the process time consuming.

Although the software and scanner used in this work are inexpensive, there are two barriers that may need to be overcome for any center wishing to implement digital design and fabrication in the manner presented. First is the investment in learning that must be done to fully utilize the techniques. This is significant and should not be underestimated. At our center, the efficiencies gained by eliminating the appointments and manual labor required for our previous methods offset this time investment, not taking into account the improvement of our finished accessories. Second, there may be restrictions on the use of such software and hardware in other jurisdictions—local regulations would need to be followed, and the local ethics board may need to be consulted. The use of MeshMixer and the 3D scanner in this work does not qualify the process for approval within Canada or elsewhere.

A reasonable, initial set of tolerances can be derived from the QA results. For our printer, material and process we can achieve an average CT number equal to the reference value within 30 HU for a wide variety of print shapes and sizes. We can also achieve better uniformity than that of the hydrogel sheet we traditionally use. The mean shape error is routinely <0.5 mm, and errors occurring on surfaces in contact with the patient up to 1.5 mm had no perceived impact on fit. Boluses that do not meet these standards can be further assessed to see if re‐printing is warranted.

Future work will include development of clinically meaningful QA tolerances for our boluses, which would likely be modality and site specific. We would also like to confirm the reproducibility in placement of our digitally designed accessories—this has not yet been done because it would require changing set up and IGRT procedures. It seems reasonable that reproducibility should increase, but there may be other factors (eg, patient‐related changes) that negate some of this benefit.

## 6 Conclusions

This study demonstrates that 3D scanning, digital design, and 3D printing can be safely and effectively utilized to modernize bolus fabrication. The patients included here spanned a broad range of disease and treatment sites. To ensure this new technology was implemented safely, QA tests were developed which are quick, quantitative, and thorough. Our process can be easily adopted by other centers since there is no reliance on equipment or software that is prohibitively costly. Both quantitative and qualitative data presented suggest that this design and fabrication process will enhance treatments requiring accessories in body regions with complex contours.

## Conflict of Interest

No conflicts of interest.
